# Supplementary material for: Effect of dialysate buffer practices on serum parathyroid hormone concentrations in real-life french patients receiving hemodialysis
Source: PLoS One. 2026 Apr 13;21(4):e0345776. doi: 10.1371/journal.pone.0345776 (PMC13075673; doi:10.1371/journal.pone.0345776)
Supplement: S1 File — Table S2: Variance inflation factors for covariates in the LMM. Fig S1: Imputed versus observed distributions of clinical variables. The red line represents the distribution of the observed data, and the black lines represent the distributions across the imputed datasets; the close overlap supports consistency between observed and imputed values. Fig S2: Mean convergence of imputed values across iterations. The x-axis represents the number of iterations; stability across iterations (no drift) supports convergence of the imputation procedure. (DOCX) [file pone.0345776.s001.docx]

**Table S1.**

| **Variable** | **Percentage of total missing data** |
| --- | --- |
| PTH | 0% |
| Age | 0% |
| Sexe | 0% |
| BMI | 1.0% |
| CCI | 28.9% |
| Site | 0% |
| 25OHD | 49.2% |
| Albumin | 30.7% |
| Calcium | 10.2% |
| Chlore | 12.5% |
| CRP | 64.2% |
| Glucose | 16.4% |
| Hemoglobin | 11.8% |
| Serum Magnesium | 54.5% |
| Dialysate calcium concentration | 0% |
| ALP | 32.2% |
| BAP | 61.2% |
| Bicarbonates | 12.7% |
| Sodium | 12.5% |
| Phosphate | 10.5% |
| Kt/V | 5.2% |
| Dialysis_prescription | 0% |
| Type of CKD | 0% |
| Type of dialysis | 0% |
| Cramps in sessions before PTH | 0% |
| Hypocalcemia before PTH | 0% |
| Hypercalcemia before PTH | 0% |
| Systolic hypotension before PTH | 0% |
| Hypomagnesemia before PTH | 0% |
| Calcimimetic | 0% |
| Calcium salts | 0% |
| Phosphate binders | 0% |
| Native vitamin D | 0% |
| Alfacalcidol | 0% |
| Magnesium | 0% |
| Corticosteroids | 0% |
| Denosumab | 0% |
| Hypertension | 0% |
| Diabetes | 0% |
| Cerebrovascular disease | 0% |
| Cancer | 0% |
| Hepatitis B | 0% |
| Hepatitis C | 0% |
| Hepatic Cirrhosis | 0% |
| Peripheral artery disease | 0% |
| Dialysate type | 0% |

**Figure S1.**


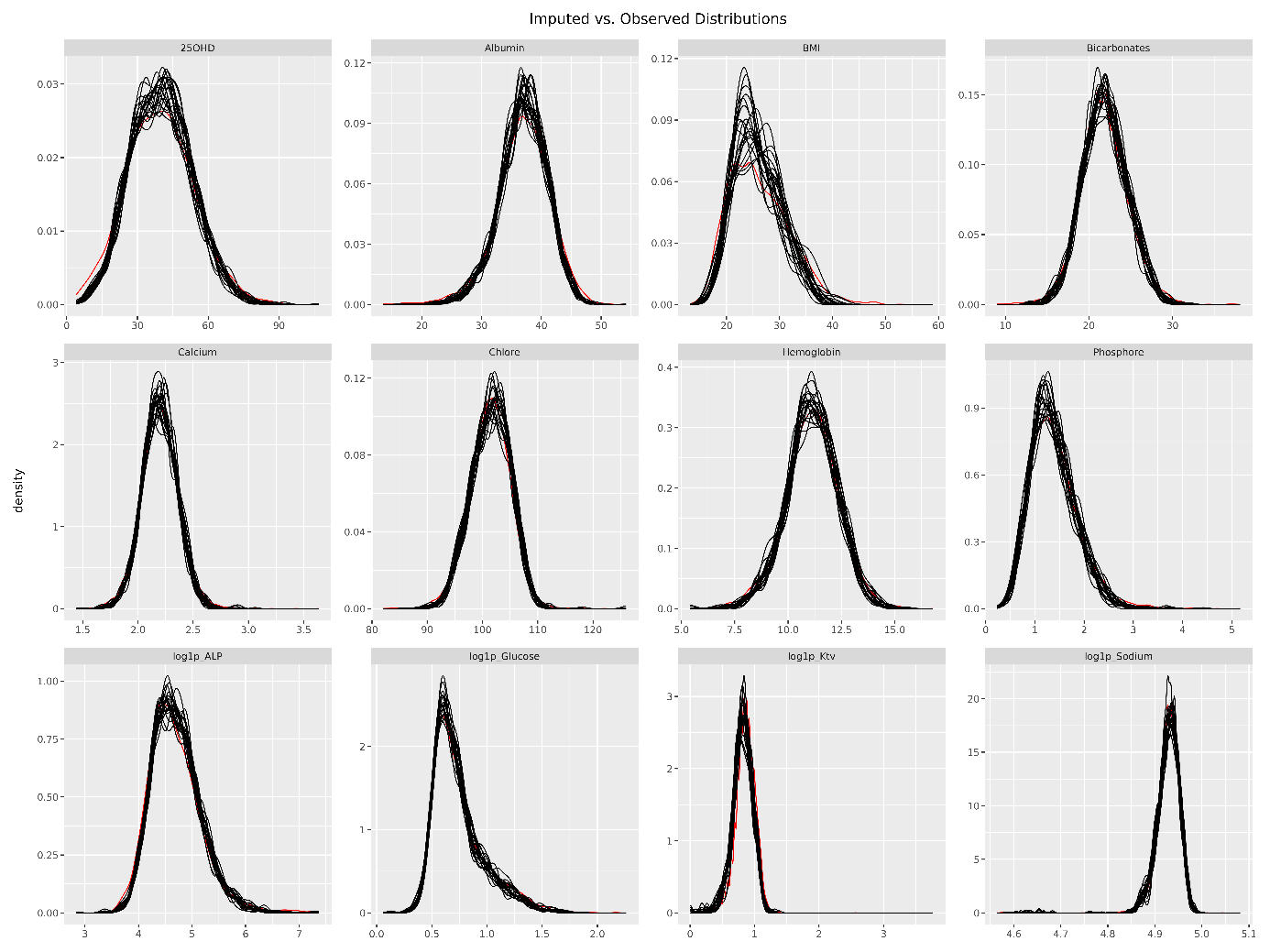


**Figure S2.**


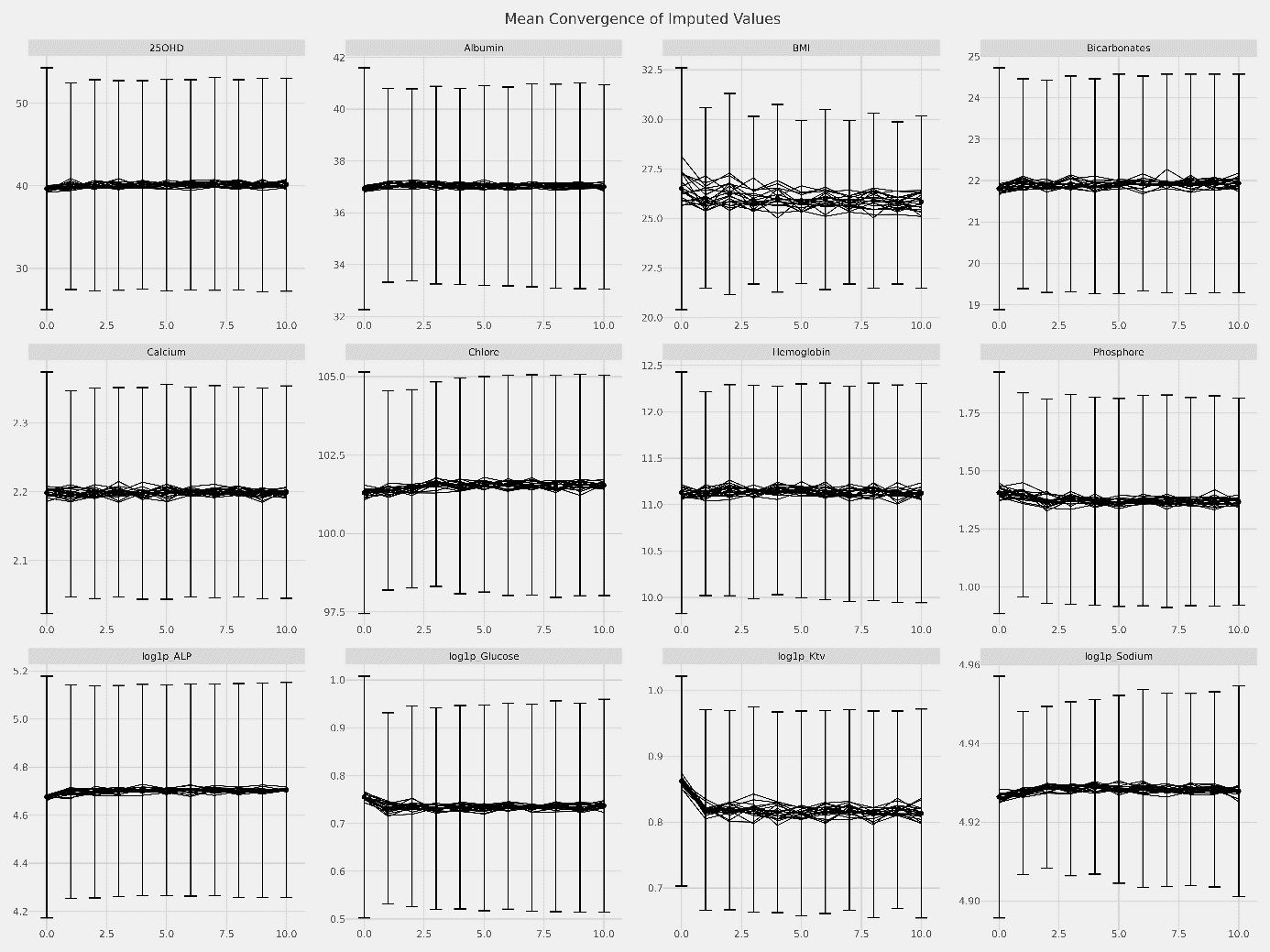


**Table S2.**

| Variable | VIF |
| --- | --- |
| Age | 3.98 |
| CCI | 3.72 |
| Chlore | 1.79 |
| Diabetes | 1.67 |
| Sodium | 1.60 |
| Glucose | 1.53 |
| Phosphate | 1.52 |
| Albumin | 1.51 |
| ALP | 1.41 |
| Bicarbonates | 1.38 |
| Native vitamin D | 1.37 |
| Phosphate binders | 1.34 |
| Calcium | 1.25 |
| Peripheral artery disease | 1.25 |
| BMI | 1.24 |
| 25OHD | 1.22 |
| Calcium salts | 1.22 |
| Alfacalcidol | 1.21 |
| Dialysis prescription | 1.19 |
| Hemoglobin | 1.19 |
| Calcimimetic | 1.18 |
| HepatitisB | 1.12 |
| Cerebrovascular disease | 1.12 |
| Hypocalcemia before PTH | 1.11 |
| Hypertension | 1.11 |
| Cancer | 1.11 |
| Dialysate calcium concentration | 1.10 |
| Ktv | 1.09 |
| Magnesium | 1.09 |
| Hypercalcemia before PTH | 1.09 |
| Denosumab | 1.09 |
| Hypomagnesemia before PTH | 1.07 |
| Hepatic Cirrhosis | 1.05 |
| Corticosteroids | 1.04 |
| Systolic hypotension before PTH | 1.04 |
| Hepatitis C | 1.04 |
| Cramps in sessions before PTH | 1.03 |
