# Supplementary material for: Effect of dialysate buffer practices on serum parathyroid hormone concentrations in real-life french patients receiving hemodialysis
Source: PLoS One. 2026 Apr 13;21(4):e0345776. doi: 10.1371/journal.pone.0345776 (PMC13075673; doi:10.1371/journal.pone.0345776)
Supplement: S2 File — (DOCX) [file pone.0345776.s002.docx]

**PROTOCOL**

**Effect of Dialysate Buffer Practices on Serum Parathyroid Hormone
Concentrations in Real-Life French Patients Receiving Hemodialysis**

| **Short title** | Dialysate buffer and PTH trajectories (AURA) |
| --- | --- |
| **Protocol version** | 1.0 |
| **Date** | 27 June 2024 |
| **Sponsor / Data controller** | AURA Paris (Association pour l’Utilisation du Rein Artificiel en Région Parisienne) |
| **Coordinating investigator** | Pablo Urena |
| **Confidentiality** | Internal scientific document. Not for public distribution without authorization. |

Table of contents

[Table of contents 2](#_Toc220506385)

[Abbreviations 3](#_Toc220506386)

[Synopsis 3](#_Toc220506387)

[1. Background and rationale 4](#_Toc220506388)

[2. Objectives 4](#_Toc220506389)

[2.1 Primary objective 4](#_Toc220506390)

[2.2 Secondary objectives 4](#_Toc220506391)

[3. Study design 5](#_Toc220506392)

[3.1 Study period and follow-up framework 5](#_Toc220506393)

[3.2 Time scale and baseline definition 5](#_Toc220506394)

[3.3 Unit of analysis and clustering 5](#_Toc220506395)

[4. Study setting 5](#_Toc220506396)

[5. Study population 6](#_Toc220506397)

[5.1 Inclusion criteria 6](#_Toc220506398)

[5.2 Exclusion criteria 6](#_Toc220506399)

[5.3 Incident and prevalent patients 6](#_Toc220506400)

[5.4 Cohort entry, follow-up, and end of observation 6](#_Toc220506401)

[6. Data sources and access 6](#_Toc220506402)

[6.1 Data source 6](#_Toc220506403)

[6.2 Date of access and de-identification 7](#_Toc220506404)

[6.3 Data extraction, linkage, and dataset structure 7](#_Toc220506405)

[6.4 Harmonisation, units, and derived variables 7](#_Toc220506406)

[6.5 Data quality, plausibility rules, and handling of outliers 7](#_Toc220506407)

[6.6 Audit trail and documentation 7](#_Toc220506408)

[7. Study variables 8](#_Toc220506409)

[7.1 Exposure: dialysate buffer type (time-updated) 8](#_Toc220506410)

[7.2 Dialysate calcium concentration (time-updated covariate) 8](#_Toc220506411)

[7.3 Outcomes 8](#_Toc220506412)

[Primary outcome 8](#_Toc220506413)

[Secondary outcomes 8](#_Toc220506414)

[7.4 PTH categories (descriptive) 8](#_Toc220506415)

[7.5 Medication exposure variables (when available) 8](#_Toc220506416)

[7.6 Other dialysis prescription parameters (descriptive) 8](#_Toc220506417)

[7.7 Timing of measurements and alignment rules 9](#_Toc220506418)

[8. Covariates (potential confounders) 9](#_Toc220506419)

[8.1 Baseline covariates 9](#_Toc220506420)

[8.2 Time-updated covariates 9](#_Toc220506421)

[9. Study size 9](#_Toc220506422)

[10. Statistical analysis plan 9](#_Toc220506423)

[10.1 General principles 9](#_Toc220506424)

[10.2 Descriptive analysis 10](#_Toc220506425)

[10.3 Missing data 10](#_Toc220506426)

[10.4 Primary longitudinal modeling (PTH trajectories) 10](#_Toc220506427)

[10.5 Effect estimates and interpretation 10](#_Toc220506428)

[10.6 Secondary analyses 10](#_Toc220506429)

[10.7 Model diagnostics and statistical assumptions 10](#_Toc220506430)

[10.8 Presentation of results 10](#_Toc220506431)

[10.9 Multiplicity and interpretation 11](#_Toc220506432)

[11. Bias, confounding, and limitations 11](#_Toc220506433)

[12. Data management and security 11](#_Toc220506434)

[12.1 Data flow and roles 11](#_Toc220506435)

[12.2 Data cleaning and engineering steps 12](#_Toc220506436)

[12.3 Reproducibility and secure storage 12](#_Toc220506437)

[13. Ethical considerations 12](#_Toc220506438)

[14. Dissemination 12](#_Toc220506439)

[15. Project timeline 12](#_Toc220506440)

[16. References 13](#_Toc220506441)

[Appendix A. Table and figure shells 13](#_Toc220506442)

[Appendix B. Planned reporting checklist (STROBE/RECORD) 15](#_Toc220506443)

# Abbreviations

| **ALP** | Alkaline phosphatase |
| --- | --- |
| **AURA** | Association pour l’Utilisation du Rein Artificiel en Région Parisienne |
| **EHR** | Electronic health record |
| **HCl** | Hydrochloric acid |
| **HD** | Hemodialysis |
| **IRB** | Institutional Review Board |
| **LMM** | Linear mixed-effects model |
| **MAR** | Missing at random |
| **MICE** | Multiple imputation by chained equations |
| **PTH** | Parathyroid hormone (intact) |
| **SHPT** | Secondary hyperparathyroidism |

# Synopsis

| **Study design** | Longitudinal observational real-life study (retrospective EHR-based cohort). |
| --- | --- |
| **Setting** | AURA dialysis network (4 sites) in the Paris region, France. |
| **Study period** | 01 Jan 2022 to 31 Dec 2023 (routine-care follow-up). |
| **Population** | Adults receiving maintenance HD with at least one pre-dialysis intact PTH measurement. |
| **Exposure** | Dialysate buffer type (acetate, citrate, or HCl), treated as time-updated. |
| **Primary outcome** | Pre-dialysis intact PTH; modeled on the log scale. |
| **Main analysis** | Linear mixed-effects models including time, buffer type, and buffer × time interaction. |
| **Missing data** | Multiple imputation (MICE), 20 datasets; pooled with Rubin’s rules. |
| **Ethics** | Foch Hospital IRB approval (IRB00012437). Routine-care data; pseudonymized dataset. |

# 1. Background and rationale

Secondary hyperparathyroidism (SHPT) is a frequent complication of advanced chronic kidney disease and maintenance hemodialysis. Persistent disturbances in mineral metabolism contribute to elevated parathyroid hormone (PTH) concentrations, bone disease, and vascular calcification risk.

Dialysate composition is a modifiable component of routine hemodialysis care. Bicarbonate dialysate is acidified using different buffers in clinical practice, most commonly acetate, citrate, or hydrochloric acid (HCl). These buffers may influence calcium balance and acid-base status and, thereby, PTH secretion and longitudinal PTH patterns under real-life conditions.

This study leverages routinely collected electronic health record (EHR) data from the AURA dialysis network to evaluate whether dialysate buffer practices are associated with PTH trajectories over time.

In contemporary hemodialysis, bicarbonate dialysate is typically acidified to prevent precipitation of calcium and magnesium salts. Different acidifying agents are used across centers and over time, driven by local procurement, clinical preferences, and perceived tolerability. Because these choices are embedded in routine care, they represent an ideal target for real-world comparative evaluation.

Acetate is rapidly metabolized to bicarbonate but has been associated with vasodilatory effects in susceptible patients. Citrate provides an acetate-free alternative and can chelate divalent cations, potentially lowering ionized calcium during dialysis and influencing PTH secretion. Hydrochloric acid (HCl)-based formulations represent another acetate-free option; their impact on mineral balance may differ from citrate due to the absence of chelation.

PTH concentrations show substantial intra-individual variability and are measured at irregular intervals in routine practice (often monthly, but sometimes quarterly). Longitudinal mixed-effects modeling is therefore preferred over cross-sectional comparisons, as it uses all available repeated measurements while accounting for within-patient correlation and unbalanced follow-up.

This protocol is written to support transparent conduct and reporting. Although reporting checklists such as STROBE and its RECORD extension primarily apply to manuscripts, their core elements (study design, data sources, participant selection, variable definitions, and analytic methods) have been considered throughout the protocol.

# 2. Objectives

## 2.1 Primary objective

To examine the association between dialysate buffer type (acetate vs citrate vs HCl), treated as a time-updated exposure, and longitudinal changes in pre-dialysis serum intact PTH in adult hemodialysis patients.

## 2.2 Secondary objectives

- To describe and compare longitudinal patterns of routinely measured biomarkers related to mineral and bone metabolism (e.g., total calcium, phosphate, bicarbonate, ALP, albumin, 25-OH vitamin D) according to dialysate buffer practices.
- To describe annualized rates of dialysis-session adverse events captured in the EHR (e.g., hypomagnesemia when available, cramps, hypocalcemia, hypercalcemia, intradialytic hypotension) according to dialysate buffer practices.

# 3. Study design

This is a longitudinal observational real-life retrospective cohort study based on routinely collected EHR data. Repeated laboratory measurements are available over the 24-month study period (January 2022 to December 2023). The exposure (dialysate buffer type) is defined as time-updated at each laboratory time point.

## 3.1 Study period and follow-up framework

The analytic window covers 01 January 2022 to 31 December 2023. Patients contribute repeated laboratory measurements whenever they are available in routine care during this period. The number and timing of measurements can vary across individuals, reflecting standard clinical monitoring rather than a pre-specified research schedule.

Follow-up for each patient starts at the first eligible PTH measurement observed within the study window (baseline for modeling) and continues until the last available PTH measurement or the end of the study window, whichever comes first. Patients who transfer out of participating sites or discontinue hemodialysis contribute data up to their last recorded measurement.

## 3.2 Time scale and baseline definition

Time is expressed in months and derived from measurement dates recorded in the EHR. For longitudinal models, time is anchored at each patient’s baseline (first eligible PTH measurement) to provide a consistent interpretation of intercepts and trajectories. The protocol allows for irregular measurement spacing; mixed-effects models accommodate this by using the actual timing of observations.

## 3.3 Unit of analysis and clustering

The primary unit of analysis is the repeated PTH measurement. Measurements are clustered within patients, and patients are clustered within dialysis centers. Mixed-effects models include patient-level random effects to account for within-patient correlation over time. Center effects can be accounted for using fixed effects (indicator variables) or descriptive stratification, depending on model stability and completeness of center-level information.

# 4. Study setting

The study is conducted within the AURA dialysis network in the Paris region, France, including four dialysis sites:

- AURA Plaisance
- AURA Saint-Ouen
- AURA Corentin-Celton
- AURA Meaux

Patients receive maintenance hemodialysis according to standard of care. Dialysate prescriptions (including buffer type and dialysate calcium concentration) are recorded in routine clinical documentation.

All participating sites provide maintenance in-center hemodialysis under standard-of-care protocols. Dialysate prescriptions are entered into the EHR and typically remain stable over weeks to months, although changes can occur in response to biochemical targets, symptoms, or logistical factors.

Laboratory testing is performed as part of routine monitoring. PTH is measured pre-dialysis using the Abbott Architect intact PTH assay (chemiluminescent microparticle immunoassay), as reported in the manuscript. Other biochemical parameters are collected according to usual practice, most commonly on a monthly basis; in some patients, PTH monitoring may be performed quarterly.

# 5. Study population

## 5.1 Inclusion criteria

- Age 18 years or older.
- Receiving maintenance hemodialysis in one of the participating AURA sites during the study period.
- At least one pre-dialysis intact PTH measurement recorded in the EHR between 01 Jan 2022 and 31 Dec 2023.
- Dialysate buffer type documented and linkable to the timing of PTH measurements.

## 5.2 Exclusion criteria

- Age younger than 18 years.
- Missing key linkage information preventing assignment of dialysate buffer type to laboratory time points.

## 5.3 Incident and prevalent patients

Both incident and prevalent hemodialysis patients are eligible to reflect routine practice.

## 5.4 Cohort entry, follow-up, and end of observation

Cohort entry is defined operationally as the date of the first eligible PTH measurement during the study window. This choice avoids imposing assumptions about pre-2022 history while allowing each patient to contribute follow-up time according to real-life testing patterns.

The end of observation is the earliest of: (i) the last recorded PTH measurement in the EHR; (ii) the end of the study window (31 December 2023); or (iii) documented discontinuation of hemodialysis in the participating sites (e.g., transfer, transplantation, modality change) when such information is available in the EHR.

# 6. Data sources and access

## 6.1 Data source

Data are extracted from the AURA EHR system (routine-care database), including demographics, comorbidities, dialysis prescriptions, medications, laboratory results, and selected dialysis-session events.

## 6.2 Date of access and de-identification

Data were accessed for research on 18 July 2024. The analytic dataset is pseudonymized; direct identifiers are not available to analysts. Access is restricted to authorized study personnel.

## 6.3 Data extraction, linkage, and dataset structure

Data extraction is performed by authorized personnel using standardized queries against the EHR database. A unique study identifier is generated for each patient to enable linkage across tables (demographics, dialysis prescriptions, laboratory results, medications, and session events) while maintaining pseudonymization.

The analytic dataset is organized in a long format, with one row per patient per laboratory measurement date. Dialysate prescription variables are aligned to each laboratory date using time stamps recorded in the prescription module. When multiple prescriptions are recorded over time, the buffer type and dialysate calcium concentration active at the time of the laboratory measurement are assigned.

## 6.4 Harmonisation, units, and derived variables

Variables are harmonized across sites to ensure consistency of coding, units, and reference ranges. Laboratory values are stored in their original units; where multiple units are used, values are converted using documented conversion factors and checked against plausible ranges.

Derived variables include: time since baseline (months), categories of PTH for descriptive reporting, and indicator variables for key comorbidities and medication classes when available. All derivations are documented in a data dictionary and reproducible code scripts.

## 6.5 Data quality, plausibility rules, and handling of outliers

Quality checks include detection of impossible dates (e.g., laboratory dates outside the study window), duplicate records, and values outside physiologically plausible ranges. When extreme values are identified, they are verified against neighboring measurements and, when necessary, flagged for exclusion or retained with caution depending on clinical plausibility. Any exclusions are documented with a reproducible audit trail.

## 6.6 Audit trail and documentation

All steps of data extraction, cleaning, derivation, and analysis are documented. Code scripts are version-controlled, and intermediate datasets are stored with dated identifiers to ensure traceability from raw extracts to analysis-ready files.

- Range and plausibility checks for laboratory values.
- Consistency checks for dates and visit sequences.
- Duplicate detection and resolution rules.
- Verification of dialysate prescription coding and time alignment.

# 7. Study variables

## 7.1 Exposure: dialysate buffer type (time-updated)

Dialysate buffer type is categorized as acetate-acidified bicarbonate dialysate, citrate-acidified bicarbonate dialysate, or HCl-acidified bicarbonate dialysate.

Time-updated exposure definition: for each laboratory time point, the dialysate buffer used at that time is extracted from the EHR and assigned to the corresponding PTH measurement.

For descriptive summaries, patients may be assigned to the dialysate buffer used most frequently within each three-month interval.

## 7.2 Dialysate calcium concentration (time-updated covariate)

Dialysate calcium concentration (mmol/L) is treated as a time-updated covariate at each laboratory time point.

## 7.3 Outcomes

### Primary outcome

Pre-dialysis serum intact PTH measured using the Architect Intact PTH assay (Abbott). For modeling, PTH is analyzed on the log scale to improve residual distribution and enable relative interpretation.

### Secondary outcomes

Secondary outcomes include:

- Routinely measured biomarkers (where available): total calcium, phosphate, sodium, bicarbonate, ALP, albumin, 25-OH vitamin D.
- Dialysis-session adverse events captured in the EHR, reported as annualized event rates (events per patient-year): hypomagnesemia when available, cramps, hypocalcemia, hypercalcemia, intradialytic hypotension.

## 7.4 PTH categories (descriptive)

- Low: 0-120 pg/mL
- Target: 121-600 pg/mL
- High: >600 pg/mL

## 7.5 Medication exposure variables (when available)

Medications that may influence mineral metabolism will be extracted when recorded in the EHR medication module or treatment summaries. These include vitamin D and analogues, calcimimetics (e.g., cinacalcet), phosphate binders, and other therapies relevant to CKD-MBD management. Medication variables may be coded as current use at each time point (time-updated) and/or as baseline use, depending on data completeness.

## 7.6 Other dialysis prescription parameters (descriptive)

To support clinical interpretation, additional prescription parameters will be summarized when available, such as dialysate bicarbonate concentration, dialysate sodium, dialysate magnesium, treatment time, and ultrafiltration volume. These variables are not primary exposures in this protocol but may help contextualize differences between centers or prescription patterns.

## 7.7 Timing of measurements and alignment rules

PTH and other biochemical outcomes are defined as pre-dialysis values on the recorded laboratory date. When multiple laboratory results of the same analyte occur on the same day, the value closest to the dialysis session (or the pre-dialysis draw when identified) is retained. Dialysate buffer type and dialysate calcium are assigned based on the prescription active on that date.

# 8. Covariates (potential confounders)

Covariates extracted from the EHR may include:

- Demographics: age, sex.
- Dialysis-related variables: dialysis vintage, site, and routinely recorded prescription features.
- Comorbidities documented in the EHR (e.g., diabetes, cardiovascular conditions).
- Medications impacting mineral metabolism (vitamin D analogues, calcimimetics, phosphate binders) where available.
- Laboratory parameters related to mineral metabolism and nutrition/inflammation (where available).

Covariates are selected based on clinical plausibility and availability in the EHR. They are considered both as potential confounders of the association between buffer practice and PTH and as variables that improve precision of longitudinal estimates.

## 8.1 Baseline covariates

Baseline covariates include age and sex, dialysis center, and dialysis vintage when available. Comorbidities (e.g., diabetes, cardiovascular disease) are identified using problem lists, diagnosis codes, or structured comorbidity fields in the EHR at or before cohort entry.

## 8.2 Time-updated covariates

When data are sufficiently complete, time-updated covariates can include dialysate calcium concentration, concurrent medications relevant to CKD-MBD, and contemporaneous laboratory values such as phosphate, calcium, bicarbonate, albumin, and ALP. Time-updated covariates are aligned to measurement dates using the same alignment rules described for exposures.

# 9. Study size

This retrospective EHR cohort includes all eligible patients during the study window. No prospective sample size calculation is required.

# 10. Statistical analysis plan

## 10.1 General principles

- Two-sided statistical tests with alpha = 0.05.
- Analyses conducted on multiply imputed datasets and pooled using Rubin’s rules.
- Statistical software: R / RStudio and Python.

## 10.2 Descriptive analysis

Baseline demographic, clinical, and laboratory characteristics are summarized by dialysate buffer group using counts and percentages for categorical variables and mean (SD) or median (Q1-Q3) for continuous variables as appropriate.

## 10.3 Missing data

Missing covariate data are handled using Multiple Imputation by Chained Equations (MICE) under a Missing At Random (MAR) assumption. Variables with less than 50% missingness are eligible for imputation. Twenty imputed datasets are generated; analyses are performed within each dataset and pooled with Rubin’s rules.

## 10.4 Primary longitudinal modeling (PTH trajectories)

Linear mixed-effects models (LMMs) are used to assess the association between dialysate buffer type and longitudinal PTH evolution.

Model specification (conceptual):

- Outcome: log(PTH).
- Fixed effects: time, dialysate buffer type (time-updated), and buffer × time interaction.
- Random effects: patient-level random effects to account for repeated measures within individuals.
- Adjustment: clinically relevant covariates as specified in the manuscript-aligned approach.

## 10.5 Effect estimates and interpretation

Adjusted differences on the log scale are back-transformed to provide relative differences with 95% confidence intervals.

## 10.6 Secondary analyses

Secondary and sensitivity analyses may be conducted to assess the robustness of the primary results and explore potential effect modification by key dialysis-related characteristics. Dialysis modality (conventional HD vs online HDF) may be evaluated as a potential effect modifier of the dialysate effect over time, using interaction and/or stratified approaches within the linear mixed-effects framework. These analyses will be considered exploratory.

## 10.7 Model diagnostics and statistical assumptions

Model diagnostics will be performed to assess the adequacy of linear mixed-effects assumptions, including inspection of residual distributions on the log scale, assessment of influential observations, and evaluation of random-effects distributions. Where appropriate, alternative variance structures or transformations will be considered for secondary biomarkers to improve model fit while preserving interpretability.

## 10.8 Presentation of results

Results will be presented as adjusted ratios (or percent differences) after back-transformation from the log scale, with 95% confidence intervals. Predicted PTH trajectories will be plotted over time by dialysate buffer group using model-based predictions, facilitating clinical interpretation of longitudinal differences. Descriptive figures will summarize prescription patterns (buffer type and dialysate calcium) over time.

## 10.9 Multiplicity and interpretation

The primary inference focuses on the association between dialysate buffer type and longitudinal PTH trajectories. Secondary outcomes are interpreted descriptively or as exploratory analyses. Given the observational nature and multiple comparisons across biomarkers, emphasis will be placed on effect sizes, confidence intervals, and clinical plausibility rather than p-values alone.

# 11. Bias, confounding, and limitations

As an observational real-world study, key limitations include:

- Confounding by indication: buffer type and dialysate calcium may be prescribed based on patient characteristics and clinician preference.
- Residual confounding by unmeasured factors (e.g., ionized calcium, diet, adherence, center-level practices).
- Heterogeneous laboratory monitoring frequency (monthly vs quarterly PTH) across patients.
- Missing data: multiple imputation addresses missingness under MAR, which may not hold in all situations.

Mixed-effects modeling leverages within-patient repeated measures and accounts for correlation across time points.

Additional considerations include center-level practice variation and changes in procurement over time. Center effects may capture unmeasured site-specific factors such as laboratory workflows, prescription culture, and patient case-mix.

Because buffer type can change over time, time-updated exposure assignment reduces misclassification compared with assigning a single baseline buffer. However, time-varying confounding (e.g., prescription changes triggered by evolving biochemistry) cannot be fully ruled out in an observational design and will be acknowledged in interpretation.

# 12. Data management and security

Data processing complies with GDPR and applicable French regulations for secondary use of health data. The analytic dataset is pseudonymized and stored on secure servers with controlled access.

Key measures include:

- Role-based access control and audit trails for data access.
- Secure storage and encrypted backups per institutional policies.
- Reporting of aggregated results only, avoiding small-cell disclosure.

## 12.1 Data flow and roles

The data management workflow includes extraction of raw EHR tables, creation of an analysis-ready dataset, and storage of derived outputs. Responsibilities are shared between the clinical data management team (extraction and pseudonymization) and the analysis team (derivations, quality checks, and statistical programming).

## 12.2 Data cleaning and engineering steps

Key processing steps include: (i) harmonizing patient identifiers and visit dates; (ii) resolving duplicates and selecting one value per analyte per day; (iii) aligning prescriptions to laboratory dates; (iv) generating derived variables (time since baseline, PTH categories); and (v) producing quality-control summaries (missingness tables, range checks, and distributions by center).

## 12.3 Reproducibility and secure storage

All analysis code is stored in a version-controlled repository with dated releases corresponding to protocol versions. Intermediate datasets and outputs are stored on secure institutional servers, with access limited to authorized personnel. Only aggregated results will be shared outside the data controller environment.

# 13. Ethical considerations

The study is non-interventional and uses routine-care data collected during standard hemodialysis follow-up. Patient information and non-opposition procedures follow local policies.

Ethics committee approval: Foch Hospital Institutional Review Board (IRB00012437), favorable opinion dated 17 July 2024.

This study involves secondary use of routinely collected health data. Risks to confidentiality are mitigated through pseudonymization, restricted access, and reporting of aggregated results. For publication and open science requirements, an anonymized extract of the analysis dataset (without direct identifiers, facility names, or exact dates) will be made publicly available as Supporting Information.

Information on the reuse of data for research is provided to patients according to AURA procedures. Patients may exercise their right to object (opt-out) as permitted by applicable regulations. Any opt-out requests will be implemented in the extraction process when feasible.

# 14. Dissemination

Findings will be disseminated through peer-reviewed publication and scientific presentations. Dissemination will use aggregated results and comply with privacy and data protection requirements.

Manuscript preparation will follow STROBE reporting guidance, with attention to the RECORD extension for studies using routinely collected health data. This includes transparent description of data sources, coding and linkage, participant selection, and handling of missing data.

Authorship will follow ICMJE recommendations. The analysis code and non-identifiable summary outputs may be shared with co-authors to support reproducibility, subject to data controller policies.

# 15. Project timeline

| **Milestone** | **Date / period** |
| --- | --- |
| Ethics committee approval (Foch Hospital IRB) | 17 July 2024 |
| Data access | 18 July 2024 |
| Data management / cleaning / engineering | Until 30 October 2024 |
| Statistical analysis | Until 30 January 2025 |
| Manuscript writing | Until 30 April 2025 |

# 16. References

Reporting guidance: STROBE statement and RECORD extension for studies using routinely collected health data.

Assay information: Abbott Architect Intact PTH assay (chemiluminescent microparticle immunoassay).

# Appendix A. Table and figure shells

A.1 Table 1 shell: Baseline characteristics by dialysate buffer group

| **Characteristic** | **Total** | **Acetate** | **Citrate** | **HCl** |
| --- | --- | --- | --- | --- |
| N | — | — | — | — |
| Age, years | — | — | — | — |
| Male sex, n (%) | — | — | — | — |
| Dialysis vintage, years | — | — | — | — |
| Diabetes, n (%) | — | — | — | — |
| Baseline PTH, pg/mL (median [IQR]) | — | — | — | — |
| Dialysate calcium, mmol/L | — | — | — | — |

A.2 Table 2 shell: Primary mixed-effects model results (log(PTH))

| **Parameter** | **Estimate** | **95% CI** | **P value** |
| --- | --- | --- | --- |
| Citrate vs acetate (main effect) | — | — | — |
| HCl vs acetate (main effect) | — | — | — |
| Time (per month) | — | — | — |
| Citrate × time | — | — | — |
| HCl × time | — | — | — |

Note: Estimates are expressed as ratios or percent differences after back-transformation from the log scale.

A.3 Figure shells

- Figure 1. Serum PTH levels by dialysate type over 24-month follow-up.
- Figure 2. Estimated PTH trajectories by dialysate for total patients (model-based, back-transformed).

# Appendix B. Planned reporting checklist (STROBE/RECORD)

For the manuscript, we will use STROBE (cohort studies) and the RECORD extension as a structured checklist to ensure that all key reporting elements are addressed. This includes (non-exhaustively): a clear description of the EHR data source, codes and algorithms used to define exposures and outcomes, linkage methods across EHR tables, participant selection flow, handling of missing data, and availability of analytic code and materials where permitted.

Key items to document explicitly in the manuscript:

- Definition of the study population and selection criteria, including numbers at each step (flow diagram if appropriate).
- Precise operational definitions of dialysate buffer exposure and alignment to laboratory dates.
- Laboratory assays, units, and any harmonisation or conversion rules.
- Missing data patterns and the multiple imputation strategy (including variables included in the imputation model).
- Statistical model specification, software, and reproducibility resources (code availability as permitted).
- Limitations specific to routinely collected data (misclassification, unmeasured confounding, changes in practice over time).
