# Supplementary material for: Effect of dialysate buffer practices on serum parathyroid hormone concentrations in real-life french patients receiving hemodialysis
Source: PLoS One. 2026 Apr 13;21(4):e0345776. doi: 10.1371/journal.pone.0345776 (PMC13075673; doi:10.1371/journal.pone.0345776)
Supplement: S3 File — (DOCX) [file pone.0345776.s003.docx]

**Statistical Analysis Plan**

Effect of Dialysate Buffer Practices on Serum Parathyroid Hormone (PTH)
in Real-Life French Patients Receiving Hemodialysis

**Version 1.0 – 23 July 2024**
Prepared by: Moustafa Naja, PhD (Biostatistician)

# Document history

| Version | Date | Summary of changes |
| --- | --- | --- |
| 1.0 | 2024-07-23 | Initial SAP created from the final study manuscript and dataset structure. |

# Abbreviations

- **ALP:** Total alkaline phosphatase
- **CCI:** Charlson Comorbidity Index
- **CKD-MBD:** Chronic kidney disease-mineral and bone disorder
- **EHR:** Electronic health record
- **HD:** Hemodialysis
- **HDF:** Hemodiafiltration
- **LMM:** Linear mixed-effects model
- **LRT:** Likelihood-ratio test
- **MAR:** Missing at random
- **MICE:** Multiple imputation by chained equations
- **PTH:** Parathyroid hormone

# 1. Background and rationale

Dialysate buffer composition (acetate, citrate, or hydrochloric acid) may influence calcium balance and acid-base exposure during hemodialysis, potentially affecting parathyroid hormone (PTH) secretion and the course of secondary hyperparathyroidism in routine practice. Because dialysate prescription is not randomized in real life, analyses must account for baseline case-mix and time-updated factors that also shape PTH.

# 2. Study objectives and estimands

## 2.1 Primary objective

To compare longitudinal trajectories of pre-dialysis serum PTH across dialysate buffer types (acetate as reference, citrate, and hydrochloric acid) over up to 24 months of follow-up.

### Primary estimand

The primary estimand is the difference in the annual rate of change (slope) in log-transformed PTH between dialysate groups, captured by the Dialysate × Time interaction in a linear mixed-effects model, conditional on measured confounders.

## 2.2 Secondary objectives

- To compare longitudinal patterns in other CKD-MBD biomarkers (e.g., total calcium, phosphate, ALP, 25-OH-D, albumin, sodium, bicarbonate).
- To describe and compare the annualized rate of selected adverse events documented in the EHR (hypomagnesemia, cramps, hypocalcemia, hypercalcemia, hypotension).
- To describe transitions between clinically meaningful PTH categories over time.

## 2.3 Exploratory objectives

- To assess robustness of findings under alternative exposure definitions (e.g., excluding dialysate switchers, or using dominant dialysate over follow-up).

# 3. Study design and data source

This is a longitudinal observational real-life study using routinely collected clinical data extracted from the AURA Paris nephrology network EHR (Medial, Nantes, France). The study period spans January 1, 2022 to December 31, 2023. Data were accessed for research on July 18, 2024. The analysis dataset is pseudonymized for research use; analyses do not use direct identifiers.

## 3.1 Setting

Four dialysis sites within the AURA network contributed data. Dialysate prescription reflects local routine practice and is not randomized.

## 3.2 Target sample size

The cohort includes all eligible adult patients treated in the participating sites during the study period. Based on the final extract used for the manuscript analyses, the expected sample size is approximately 876 patients, with exposure distributed across the three dialysate buffer types and allowing for dialysate switching over time.

## 3.3 Ethical and regulatory considerations

Non-opposition to the use of patient-level data for research was obtained. The study received Institutional Review Board approval (Foch Hospital, Suresnes, France; IRB00012437) and was conducted in accordance with the Declaration of Helsinki.

# 4. Study population

## 4.1 Inclusion criteria

- Adults aged 18 years or older receiving maintenance HD or HDF in participating AURA sites.
- At least one pre-dialysis PTH measurement recorded during the study period.
- Dialysate buffer type recorded (acetate, citrate, or hydrochloric acid).

## 4.2 Exclusion criteria

- Patients with missing dialysate buffer information at all time points.
- Measurements with implausible or clearly erroneous laboratory values that cannot be resolved after data checks (see Section 7).

## 4.3 Index date and follow-up

The index date is defined as the first available pre-dialysis PTH measurement within the study period (baseline). Follow-up is defined from baseline through up to 24 months, censored at the last available PTH measurement, death, kidney transplantation, transfer out, or end of the extraction window (whichever occurs first). Time is analyzed as continuous years since baseline.

# 5. Exposure and treatment patterns

## 5.1 Dialysate buffer type

Dialysate buffer type is categorized as acetate-based, citrate-based, or hydrochloric acid-based bicarbonate dialysate. Because patients may switch dialysate over time, dialysate buffer is treated as a time-varying exposure. For descriptive analyses of category transitions, patients are assigned to the dialysate used most frequently during each 3-month interval.

## 5.2 Dialysate calcium concentration

Dialysate calcium concentration (mmol/L) is treated as a time-updated covariate and analyzed in categories consistent with routine prescriptions (e.g., <=1.50, 1.65, 1.75).

# 6. Outcomes, covariates, and derived variables

## 6.1 Primary outcome

Repeated pre-dialysis serum PTH measurements (pg/mL) collected routinely (typically monthly) are the primary outcome. PTH is log-transformed for modeling to reduce skewness and improve residual normality.

## 6.2 Secondary outcomes

- Repeated biomarkers (monthly where available): total calcium, phosphate, ALP (and/or BAP where available), 25-OH vitamin D, albumin, sodium, chloride, bicarbonate, hemoglobin, CRP, and others recorded in the EHR.
- EHR-recorded adverse events assessed on a 3-monthly basis (presence/absence at least once in the interval): hypomagnesemia, cramps, hypocalcemia, hypercalcemia, and hypotension.

## 6.3 Key covariates

Candidate confounders include baseline demographics (age, sex), comorbidity burden (CCI), dialysis modality (HD/HDF), dialysis vintage, dialysis dose (Kt/V), dialysate calcium prescription, baseline PTH, and time-updated laboratory values and medication indicators.

## 6.4 Clinical thresholds and categories

- PTH categories: Low (0-120 pg/mL), Target range (121-600 pg/mL), High (>600 pg/mL).
- Hypercalcemia: serum calcium >2.65 mmol/L; Hypocalcemia: serum calcium <1.80 mmol/L.
- Hypomagnesemia: serum magnesium <0.80 mmol/L.
- Hypotension: mean systolic blood pressure <90 mmHg.

# 7. Data management and quality control

Data are extracted from the EHR into analysis-ready tables at patient and measurement levels. Quality checks include unit harmonization, duplicate removal, consistency checks across tables (e.g., medication indicators aligned to the intended 3-month windows), and screening for implausible values using clinically informed ranges. Any correction rules are documented and applied consistently across analyses.

## 7.1 Derived time variables

A continuous time variable is computed as years since baseline. For descriptive summaries, follow-up is also grouped into 3-month intervals (0-3, 3-6, ..., 21-24 months).

## 7.2 Analysis datasets

The primary longitudinal dataset is in long format, one row per patient per PTH measurement, with exposure and covariates aligned to each PTH date. Secondary datasets summarize biomarkers and adverse events within predefined intervals.

# 8. Statistical principles

All statistical tests are two-sided with a nominal alpha level of 0.05. Effect sizes are reported with 95% confidence intervals, prioritizing clinical interpretability over p-values alone. Because this is an observational study with multiple secondary analyses, secondary findings are interpreted as supportive and hypothesis-generating unless otherwise specified.

## 8.1 Handling of non-random treatment allocation

Dialysate type is prescribed according to routine practice and may reflect patient characteristics (confounding by indication). The primary analysis adjusts for baseline PTH and relevant covariates, and uses time-updated measures to reduce residual confounding. Sensitivity analyses (Section 14) evaluate robustness under alternative modeling and cohort restrictions.

# 9. Missing data

Missingness is described for each variable (overall and by dialysate group). Variables with more than 50% missing values are not used as primary covariates. Assuming data are missing at random (MAR), multiple imputation by chained equations (MICE) is used to create 20 imputed datasets. Imputation models include all variables used in the primary and key secondary analyses, as well as auxiliary variables that help predict missingness. Convergence and plausibility of imputed values are assessed through standard diagnostics (trace plots and distributional comparisons).

# 10. Descriptive analyses

Baseline characteristics are summarized by dialysate type. Categorical variables are reported as n (%), continuous variables as median (Q1-Q3) due to skewed distributions. Group comparisons use chi-square tests for categorical variables and Kruskal-Wallis tests for continuous variables, followed by Bonferroni-corrected pairwise comparisons when appropriate.

# 11. Primary analysis

## 11.1 Model specification

The primary analysis uses a linear mixed-effects model (LMM) on log(PTH) with patient-specific random intercepts and random slopes for time. Fixed effects include dialysate group (acetate reference), time (years since baseline), and the Dialysate × Time interaction. Baseline PTH and clinically relevant covariates are included; laboratory and medication covariates are incorporated as time-updated measures aligned to each PTH assessment.

## 11.2 Primary hypothesis test

The primary test evaluates whether PTH trajectories differ across dialysate groups, using a likelihood-ratio test (LRT) for the Dialysate × Time interaction. Dialysate main effects reflect adjusted differences at the model intercept (baseline) and are interpreted cautiously because baseline case-mix differs by prescription patterns.

## 11.3 Estimands and reporting

Model coefficients are exponentiated to report adjusted percentage differences in PTH. Results include (i) adjusted baseline differences, (ii) adjusted annual slope differences versus acetate, and (iii) predicted cumulative percent change at 24 months with 95% CIs. Graphical displays show estimated PTH trajectories over time by dialysate group.

## 11.4 Model diagnostics

Model adequacy is assessed via residual plots, Q-Q plots of residuals, and inspection of random-effects distributions. Influential observations are explored using standardized residuals and leverage diagnostics.

# 12. Secondary analyses

## 12.1 Other biomarkers

For selected biomarkers with sufficient longitudinal coverage, supportive analyses follow one of two approaches depending on data density: (i) interval-based summaries comparing mean values between baseline (0-3 months) and 9-12 months within each dialysate group, and (ii) mixed-effects models analogous to the primary model when repeated measures are frequent. Within-group changes use Wilcoxon signed-rank tests; between-group comparisons at 9-12 months use Mann-Whitney tests with Bonferroni correction.

## 12.2 Adverse event rates

Adverse events are summarized as annualized event rates per patient-year. Primary comparisons use chi-square tests for differences in event frequencies across groups. As a robustness check, Poisson (or negative binomial if overdispersed) regression with an offset for exposure time may be used to estimate rate ratios with 95% CIs.

## 12.3 PTH category transitions

PTH category distributions are described by 3-month intervals and dialysate assignment within each interval. Transitions are summarized descriptively; formal modeling (e.g., multinomial mixed models) is considered exploratory.

# 13. Sensitivity analysis

Dialysis modality (conventional HD vs online HDF). We forced dialysis modality into the adjusted LMM and tested a Dialysate × Time × Modality interaction to assess potential effect modification.

# 14. Multiplicity and interpretation framework

The primary analysis is pre-specified and interpreted at alpha=0.05. Secondary and exploratory analyses are not adjusted for multiplicity; findings are reported transparently and interpreted as supportive or hypothesis-generating.

# 15. Software, reproducibility, and quality assurance

Analyses are performed using Python (v3.12 or later) and R/RStudio. All code is version-controlled. Key results are reproduced from raw extracts through scripted pipelines to minimize manual processing. A second analyst review (or internal code review) is recommended for the primary model and key tables/figures prior to submission.

# 16. Planned outputs (tables and figures)

- Table 1: Baseline patient characteristics by dialysate type.
- Table 2: Pooled LMM estimates for PTH (main effects and Dialysate × Time interaction).
- Table 3: Biomarker evolution between 0-3 and 9-12 months by dialysate.
- Table 4: Annualized adverse event rates by dialysate.
- Figure 1: Serum PTH levels over time by dialysate type (descriptive).
- Figure 2: Model-estimated PTH trajectories over 24 months by dialysate.

# 17. Data availability and transparency

This study follows transparent reporting principles consistent with STROBE and the RECORD extension for routinely collected health data. In line with PLOS requirements, the data underlying the findings will be made publicly available as Supporting Information upon publication (an anonymised dataset plus a data dictionary/codebook). Before sharing, the dataset is prepared to minimize re-identification risk by removing direct identifiers and applying generalisation/suppression to key indirect identifiers (e.g., no dates of birth, no exact dialysis session dates, no center names, no internal patient identifiers; anthropometrics rounded; age presented in bands).
