# Supplementary material for: Effect of dialysate buffer practices on serum parathyroid hormone concentrations in real-life french patients receiving hemodialysis
Source: PLoS One. 2026 Apr 13;21(4):e0345776. doi: 10.1371/journal.pone.0345776 (PMC13075673; doi:10.1371/journal.pone.0345776)
Supplement: S4 File — (DOCX) [file pone.0345776.s004.docx]

**Human Participants Research Checklist**

***Complete the following if your study involved human participants or human participants’ data. These questions should be addressed for prospective and retrospective studies.***

1. Did you obtain ethics approval for this study?
   - If yes, please upload (file type “Other”) all the approval documents you received from your ethics committee to cover the entire range of the study period (i.e. the original approval document and any extension documents). Where ethics approval was obtained from more than one study location, please provide approval document(s) from all of the sites. If the original document is in another language, please also provide an English translation.

**Yes. Approved by the Institutional Review Board of Hôpital Foch (IRB00012437). See original French approval (AVIS-FOCH.pdf) and English translation attached.**

**Single IRB approval covers all participating AURA centers. No additional local approvals required.**

- - If you did not obtain ethical approval, please explain why this was not required below.

1. If you prospectively recruited human participants for the study – for example, you conducted a clinical trial, distributed questionnaires, or obtained tissues, data or samples for the purposes of this study, please report in the Methods:
   1. the day, month and year of the **start and end** of the recruitment period for this study.
   2. whether participants provided informed consent, and if so, what type was obtained (for instance, written or verbal, and if verbal, how it was documented and witnessed). If your study included minors, state whether you obtained consent from parents or guardians. If the need for consent was waived by the ethics committee, please include this information.

Please state the line number(s) in the Methods where this is reported ______

**N/A — This is a retrospective observational study using existing EHR data; no prospective recruitment.**

1. If you are reporting a retrospective study of, for example, medical records, archived samples, survey data, please report in the Methods section:

the day, month and year when the data were accessed for research purposes

**Date data were accessed: 18 July 2024**

1. whether authors had access to information that could identify individual participants during or after data collection

**Authors analyzed a pseudonymized dataset. Direct identifiers were not available to the analysis team; the re-identification key remained solely with the data controller (AURA Paris).**

Please state the line number(s) in the Methods where this is reported ______

**Non-opposition to patient-level data collection was obtained. The study was approved by the Institutional Review Board of Foch Hospital, Suresnes, France, registered under the number IRB00012437, and conducted in accordance with the ethical principles of the Declaration of Helsinki (lines 141-144)**
