# Supplementary material for: Effect of dialysate buffer practices on serum parathyroid hormone concentrations in real-life french patients receiving hemodialysis
Source: PLoS One. 2026 Apr 13;21(4):e0345776. doi: 10.1371/journal.pone.0345776 (PMC13075673; doi:10.1371/journal.pone.0345776)
Supplement: Attachment — Submitted filename: PLOS_responsestoreviewers_30012026.docx [file pone.0345776.s007.docx]

**Responses to the Editor and Reviewers**

**To the Editor**

The reviewers provided some mixed feedback about the manuscript’s methodology. However, the research question examined here remains insufficiently explored in the current literature, making the present study an important contribution and highlighting the need for dedicated prospective investigations and clinical trials. The statistical analysis used is appropriate but I kindly ask the authors to incorporate dialysis vintage into the adjusted model.

- Thank you for this suggestion. We added dialysis vintage (months) to the fully adjusted linear mixed-effects model. Vintage was not associated with serum PTH (−0.01% per month, 95% CI −0.03% to +0.01%; p = 0.426) and did not materially change the dialysate effects or the dialysate×time interaction. The updated results are reported in the Results section (“Analysis of serum PTH levels using LMM”, page 8).

In addition, the limitations section should be expanded to acknowledge the substantial proportion of missing data for several variables (up to 50%), as well as the quasi-absence of patients dialyzed with a 1.25 mmol/L calcium bath. It should also be noted in the limitations that the cohort is composed of prevalent rather than incident patients, which may introduce survivor bias and limit the generalizability of the findings.

- Agreed. We expanded the Limitations paragraph to cover (i) substantial missingness (up to 50%) and the use of multiple imputation under a missing-at-random assumption, (ii) the near-absence of 1.25 mmol/L dialysate calcium prescriptions, and (iii) the prevalent (non-incident) nature of the cohort and potential survivor bias. These points are now stated in the Discussion (Limitations, page 11).

Please provide on top of these editorial recommendations, a point-by-point response to all reviewers.

============================
Please submit your revised manuscript by Jan 16 2026 11:59PM. If you will need more time than this to complete your revisions, please reply to this message or contact the journal office at [plosone@plos.org](mailto:plosone@plos.org). When you're ready to submit your revision, log on to<https://www.editorialmanager.com/pone/> and select the 'Submissions Needing Revision' folder to locate your manuscript file.

Please include the following items when submitting your revised manuscript:

- A rebuttal letter that responds to each point raised by the academic editor and reviewer(s). You should upload this letter as a separate file labeled 'Response to Reviewers'.
- A marked-up copy of your manuscript that highlights changes made to the original version. You should upload this as a separate file labeled 'Revised Manuscript with Track Changes'.
- An unmarked version of your revised paper without tracked changes. You should upload this as a separate file labeled 'Manuscript'.

If you would like to make changes to your financial disclosure, please include your updated statement in your cover letter. Guidelines for resubmitting your figure files are available below the reviewer comments at the end of this letter.

If applicable, we recommend that you deposit your laboratory protocols in [protocols.io](http://protocols.io) to enhance the reproducibility of your results. [Protocols.io](http://Protocols.io) assigns your protocol its own identifier (DOI) so that it can be cited independently in the future. For instructions see: [https://journals.plos.org/plosone/s/submission-guidelines#loc-laboratory-protocols](https://track.editorialmanager.com/CL0/https:%2F%2Fjournals.plos.org%2Fplosone%2Fs%2Fsubmission-guidelines%23loc-laboratory-protocols/1/010f019ae2221535-cf4a122f-20bd-44ab-bd24-c205d16739b7-000000/F_s7BO4dBV6fPWeGXHPR_GCsz9jsZav9QDTl5ynR_Zs=239). Additionally, PLOS ONE offers an option for publishing peer-reviewed Lab Protocol articles, which describe protocols hosted on [protocols.io](http://protocols.io). Read more information on sharing protocols at [https://plos.org/protocols?utm_medium=editorial-email&utm_source=authorletters&utm_campaign=protocols](https://track.editorialmanager.com/CL0/https:%2F%2Fplos.org%2Fprotocols%3Futm_medium=editorial-email%26utm_source=authorletters%26utm_campaign=protocols/1/010f019ae2221535-cf4a122f-20bd-44ab-bd24-c205d16739b7-000000/pkfUX3ysi6ROBi6el8I54x0mEVJySA1-4muCvxTnzjo=239).

We look forward to receiving your revised manuscript.

Kind regards,

Mabel Aoun, MD, MPH
Academic Editor
PLOS ONE

Journal Requirements:

When submitting your revision, we need you to address these additional requirements.

1. Please ensure that your manuscript meets PLOS ONE's style requirements, including those for file naming. The PLOS ONE style templates can be found at

<https://journals.plos.org/plosone/s/file?id=wjVg/PLOSOne_formatting_sample_main_body.pdf> and

[https://journals.plos.org/plosone/s/file?id=ba62/PLOSOne_formatting_sample_title_authors_affiliations.pdf](https://track.editorialmanager.com/CL0/https:%2F%2Fjournals.plos.org%2Fplosone%2Fs%2Ffile%3Fid=ba62%2FPLOSOne_formatting_sample_title_authors_affiliations.pdf/1/010f019ae2221535-cf4a122f-20bd-44ab-bd24-c205d16739b7-000000/eqk3gawsH7AWEgR6OE93Hk1xosOLzO_fzDcn2O3RBKE=239)

2. In the online submission form, you indicated that “The study protocol, statistical analysis plan, and all data presented in this article are available to other researchers on request.”

All PLOS journals now require all data underlying the findings described in their manuscript to be freely available to other researchers, either 1. In a public repository, 2. Within the manuscript itself, or 3. Uploaded as supplementary information.This policy applies to all data except where public deposition would breach compliance with the protocol approved by your research ethics board. If your data cannot be made publicly available for ethical or legal reasons (e.g., public availability would compromise patient privacy), please explain your reasons on resubmission and your exemption request will be escalated for approval.

- Thank you for highlighting the PLOS Data Availability Policy. We have now prepared a fully anonymised dataset underlying the findings (with removal of direct identifiers and generalisation/suppression of key indirect identifiers to minimise re-identification risk under GDPR and French data-protection requirements). The anonymised dataset underlying the findings of this study is provided as Supporting Information files.

3. When completing the data availability statement of the submission form, you indicated that you will make your data available on acceptance. We strongly recommend all authors decide on a data sharing plan before acceptance, as the process can be lengthy and hold up publication timelines. Please note that, though access restrictions are acceptable now, your entire data will need to be made freely accessible if your manuscript is accepted for publication. This policy applies to all data except where public deposition would breach compliance with the protocol approved by your research ethics board. If you are unable to adhere to our open data policy, please kindly revise your statement to explain your reasoning and we will seek the editor's input on an exemption. Please be assured that, once you have provided your new statement, the assessment of your exemption will not hold up the peer review process.

- We agree and have revised our Data Availability Statement accordingly. The anonymised individual-level dataset underlying the results is already provided as Supporting Information and will be freely accessible upon publication, consistent with PLOS.

4. Please amend the manuscript submission data (via Edit Submission) to include author Moustafa Naja, Minh Hoang Tran, Martine Cohen Solal, Jordi Bover, Anne Kolko, Pascal Seris, Ibrahim Farah, Natalia Alençar-de Pinho, Ziad A. Massy, Charles Chazot.

- We have amended the submission data (Edit Submission) to include the full author list: Moustafa Naja, Minh Hoang Tran, Martine Cohen Solal, Jordi Bover, Anne Kolko, Pascal Seris, Ibrahim Farah, Natalia Alençar-de Pinho, Ziad A. Massy, and Charles Chazot.

5. Please ensure that you refer to Figure 1 and 2 in your text as, if accepted, production will need this reference to link the reader to the figure.

- Figures 1 and 2 are already explicitly cited in the main text at the relevant points (Results), using “(Figure 1)” and “Figure 2…”. We have double-checked to ensure each figure is referenced in-text for production linking.

6. If the reviewer comments include a recommendation to cite specific previously published works, please review and evaluate these publications to determine whether they are relevant and should be cited. There is no requirement to cite these works unless the editor has indicated otherwise.

- No specific reviewer recommendations to cite particular previously published works were provided; therefore, no additional citations were added.

[Note: HTML markup is below. Please do not edit.]

Reviewers' comments:

Reviewer's Responses to Questions

**Comments to the Author**

1. Is the manuscript technically sound, and do the data support the conclusions?

The manuscript must describe a technically sound piece of scientific research with data that supports the conclusions. Experiments must have been conducted rigorously, with appropriate controls, replication, and sample sizes. The conclusions must be drawn appropriately based on the data presented.

Reviewer #1: No

Reviewer #2: Partly

Reviewer #3: Yes

Reviewer #4: Yes

Reviewer #5: Partly

2. Has the statistical analysis been performed appropriately and rigorously?

Reviewer #1: Yes

Reviewer #2: I Don't Know

Reviewer #3: I Don't Know

Reviewer #4: Yes

Reviewer #5: No

3. Have the authors made all data underlying the findings in their manuscript fully available?

The [PLOS Data policy](http://track.editorialmanager.com/CL0/http:%2F%2Fwww.plosone.org%2Fstatic%2Fpolicies.action%23sharing/1/010f019ae2221535-cf4a122f-20bd-44ab-bd24-c205d16739b7-000000/msk61ol9skJPVSnyNzo8yrKQqxAK0UV6zMLw45O0Gb4=239) requires authors to make all data underlying the findings described in their manuscript fully available without restriction, with rare exception (please refer to the Data Availability Statement in the manuscript PDF file). The data should be provided as part of the manuscript or its supporting information, or deposited to a public repository. For example, in addition to summary statistics, the data points behind means, medians and variance measures should be available. If there are restrictions on publicly sharing data—e.g. participant privacy or use of data from a third party—those must be specified.

Reviewer #1: No

Reviewer #2: Yes

Reviewer #3: Yes

Reviewer #4: Yes

Reviewer #5: Yes

4. Is the manuscript presented in an intelligible fashion and written in standard English?

PLOS ONE does not copyedit accepted manuscripts, so the language in submitted articles must be clear, correct, and unambiguous. Any typographical or grammatical errors should be corrected at revision, so please note any specific errors here.

Reviewer #1: No

Reviewer #2: Yes

Reviewer #3: Yes

Reviewer #4: Yes

Reviewer #5: No

5. Review Comments to the Author

Please use the space provided to explain your answers to the questions above. You may also include additional comments for the author, including concerns about dual publication, research ethics, or publication ethics. (Please upload your review as an attachment if it exceeds 20,000 characters)

Reviewer #1: The authors attempt to compare 3 different types of dialysate (hydrochloric, citrate, and acetate on mineral parameters. The main finding is a 10% decline in PTH in the hydrochloric acid group, whereas the citrate group showed a 10% increase after 24 months of follow-up. The major drawback of the study are 1) the substantial differences in the study population especially the HCl Dialysate group had the lowest PTH level at baseline, and 2) the lack of clinical outcomes associated with the decline in PTH level.
The less severe HPT can be more easily influenced by treatment or intervention compared to the more severe degree of PTH. The clinical outcomes on the decline in PTH levels such as a reduced dosage of calcimimetics or active vitamin D should be presented to confirm the significant benefit of HCl dialysate. In addition, the manuscript and all tables are difficult to follow.

- We agree on both points. Hard clinical outcomes were not available in this EHR-based dataset, and medication exposure was captured as quarterly presence/absence (no doses), so we could not assess treatment de-escalation; we now state this explicitly in the Limitations, and we report adverse-event indicators as the main available clinical correlates (Table 4).
  We also acknowledge baseline imbalances, including lower baseline PTH in the HCl group (Table 1). To address this, our mixed-effects models were adjusted for baseline PTH and other clinically relevant covariates; however, residual confounding cannot be excluded. Finally, we improved readability by adding clearer in-text pointers to Tables 1–4 and Figures 1–2.

Minor points
1.A calcium-dialysate concentration of 1.25 mmol/L was almost absent from clinical practice (<1 % of use). – How is this relevant to the study?

- Dialysate calcium concentration is a key determinant of calcium balance and PTH control, and current practice/guidelines often discuss 1.25 vs 1.50 mmol/L baths. In our cohort, 1.25 mmol/L was almost never prescribed (<1%), so we could not reliably evaluate whether a lower calcium bath modifies PTH trajectories or interacts with dialysate type. We therefore reported this as a limitation in the page 11 of the revised version of the manuscript.

2.The progressive significant decrease of PTH in the hydrochloric dialysate was accompanied by a significant rise in 25-OH-D, which increased from 39.0 ± 10.4 ng/mL at the baseline to 42.2 ± 11.3 ng/mL at one year (p<0.001), whereas serum calcium and phosphate remained unchanged in all three groups. Taken together, these observations suggest a synergistic effect, whereby hydrochloric acid dialysate, combined with optimization of vitamin D status, drives superior biochemical control. – In dialysis patients, a change in 25-OH-D level is unlikey to affect PTH level.

- We agree. Although 25-OH-D increased slightly in the hydrochloric group (statistically significant), the magnitude was small and we cannot infer a causal or “synergistic” effect on PTH in hemodialysis patients. in our adjusted LMM (which includes 25-OH-D and medication indicators), hydrochloric dialysate remained associated with a significantly steeper decline in PTH over time, suggesting that the observed trajectory difference is not explained by changes in 25-OH-D alone. We removed the synergy interpretation and now report the 25-OH-D finding as an associated observation.

Reviewer #2: The study addresses an important and clinically relevant question regarding the effect of dialysate buffer composition on PTH dynamics in HD patients. However, several major issues limit the interpretability of the findings and should be addressed before the manuscript can be considered for publication.

1. Introduction not sufficiently aligned with the study aim

The Introduction reviews the background on acetate, citrate, and hydrochloric dialysates but does not clearly articulate how dialysate composition affects calcium balance and PTH physiology, nor what differential effects the authors expected among the three groups. The rationale and the a priori hypothesis should be better specified, ideally summarizing the mechanistic links between dialysate buffer, ionized calcium, CaSR activation and PTH secretion. This clarification is essential to contextualize the results.

- Agreed. We revised the Introduction to better link buffer composition to calcium balance and PTH physiology (ionized calcium changes during dialysis, CaSR activation, and downstream PTH secretion). We also added our a priori expectation that citrate, via calcium chelation, could lower ionized calcium and stimulate PTH unless offset by a higher calcium bath, and we clarified that intradialytic ionized calcium was not measured in this real-world dataset (we therefore evaluate longitudinal pre-dialysis PTH trajectories).

2. Significant differences in dialysis modality among groups

The dialysate groups differ substantially in dialysis modality distribution, with a much higher proportion of HDF in the citrate and hydrochloric groups, while conventional HD predominates in the acetate group. Since HDF is known to influence calcium handling and PTH regulation, this represents a major potential confounder. Although statistical adjustment is mentioned, such adjustments may not fully account for this imbalance.
A stratified or interaction analysis (Dialysate × HD vs. HDF) is strongly recommended, or at minimum, the impact of modality differences should be explicitly discussed as a limitation.

- We agree that dialysis modality differed across dialysate groups and could confound PTH dynamics. In line with your suggestion, we performed a sensitivity analysis testing whether dialysis modality (HD vs HDF) modified the dialysate effect over time (per year) by adding a Dialysate × time × modality interaction to the linear mixed-effects model. We found no evidence of effect modification by modality: for citrate vs acetate, the Dialysate(citrate) × time × HDF interaction was not significant (β = 28.57 ng/L per year, 95% CI −50.31 to 107.45, p = 0.478); for hydrochloric acid vs acetate, the Dialysate(hydrochloric acid) × time × HDF interaction was also not significant (β = −30.02 ng/L per year, 95% CI −119.29 to 59.25, p = 0.510). Accordingly, dialysis modality and its interaction terms were not retained in the final parsimonious adjusted model selected by backward elimination. Nevertheless, given the observational design and non-random treatment allocation, residual confounding related to modality differences cannot be fully excluded and is acknowledged in the Limitations.

3. Differences in dialysate calcium concentration

Dialysate calcium concentration differs significantly among groups, with citrate patients predominantly receiving 1.65 mmol/L, while hydrochloric/acetate more frequently received 1.50 mmol/L. Since dialysate calcium concentration is one of the primary determinants of PTH, this difference may independently explain the observed results. Additional sensitivity analyses adjusting for dialysate Ca strata, or restricting analysis to patients with the same Ca bath, would improve interpretability.

- Agreed. dialysate calcium differed markedly by group (citrate mostly 1.65 mmol/L; hydrochloric/acetate mostly 1.50 mmol/L). Dialysate calcium was included in the adjusted LMM and was an independent predictor of PTH (Table 2). A restriction to a single calcium bath across all three dialysate types was not feasible given local prescribing patterns (notably, 1.50 mmol/L was not used with citrate and 1.65 mmol/L was specific to citrate); we therefore highlight remaining calcium-bath imbalance as a limitation and we clarified this in the discussion section in the page 11 of the revised manuscript.

Reviewer #3: Study has several strong points, notably the real-life observational design, large sample size, and two-year follow-up, which lend credibility to your findings. The use of centralized laboratory analyses and robust statistical methods adds to the reliability of the results. However, to further strengthen your study, consider addressing the biased case-mix distribution in more depth and discussing its potential impacts more thoroughly. Increasing the inclusion of incident dialysis patients and incorporating post-dialysis serum calcium measurements and total calcium balance would provide a more comprehensive understanding of mineral metabolism. Additionally, acknowledging potential unmeasured confounders will help clarify the validity of your conclusions. Overall, your research provides valuable insights into the management of SHPT and underscores the need for more controlled studies in this field.

- Thank you for these constructive suggestions. We agree that dialysate prescription was non-random and case-mix differed across groups. We expanded the Discussion/Limitations in the page 11 to discuss this potential bias, acknowledge unmeasured confounding, and clarify that our findings should be interpreted as real-world associations rather than causal effects.

We also acknowledge that our cohort is largely prevalent (not incident) hemodialysis patients, which limits generalizability to newly initiated patients. Finally, post-dialysis calcium measurements and formal calcium balance assessments were not available in this EHR-based study; we highlight this limitation and emphasize the need for prospective controlled studies including incident patients and calcium balance evaluation.

Reviewer #4: I have carefully read the article entitled
“Effect of Dialysate Buffer Practices on Serum Parathyroid Hormone Concentrations in Real-Life French Patients Receiving Hemodialysis”
by Dr. Ureña et al., in which the authors perform a large-scale analysis of laboratory data to investigate whether different dialysate solutions have distinct impacts on the evolution of serum PTH levels in patients undergoing hemodialysis.


The authors conclude that the type of dialysate has a substantial influence on medium-term PTH evolution: the citrate bath worsened PTHi levels, whereas the hydrochloric acid bath led to a decrease, using the acetate-containing bath as the reference. These results are adjusted for other influential variables, such as the use of calcium-containing phosphate binders, prescription of cinacalcet or vitamin D, and also for dialysate calcium concentration, for which they also confirm the well-known suppressive effect of increasing dialysate calcium on serum PTH levels.
As the authors show, the literature on hemodialysis using hydrochloric acid–based dialysate is very limited, and in this sense I believe the study is of interest for publication in the journal. Overall, I do not think major changes are needed. I only suggest adding a few comments.
The authors collected a large longitudinal dataset of dialysis parameters and PTHi serum levels, and performed their analysis using a mixed-effects linear model (MLM) to account for within-patient changes over time and to ultimately analyze the effect of dialysate type on the PTH slope. Given that the authors present in a table the collected variables and the percentage of missing data, I understand that the MLM analysis incorporated the time-varying covariates corresponding to each PTH measurement. It would be helpful to add a sentence clarifying explicitly that covariates were not only used at baseline but were included throughout the entire follow-up. This would make the methodology and interpretation clearer.

- Thank you for the supportive assessment and helpful suggestions. We also clarify in the results section in page 8 that citrate did not show a worsening PTH trajectory versus acetate: the Dialysate×Time interaction was not significant and the cumulative change over follow-up was non-significant.
- Agreed. We have added one sentence in the Methods Statistical analysis section in page 5 to clarify that covariates were incorporated as time-updated variables aligned with each PTH measurement (monthly biomarkers; 3-monthly medications/dialysate calcium/adverse events), rather than baseline only.

Some studies have reported that acidic pH influences the calcium–PTH set-point and could potentially alter the response to cinacalcet. Patients with more severe acidosis tend to present higher PTH levels and are less sensitive to the effects of cinacalcet and serum calcium. I believe this aspect should be mentioned in the discussion. The authors should state whether pre-dialysis serum bicarbonate was included as a covariate and whether it was significant. If it was not included, I believe it would be worthwhile adding it to rule out this potential confounder.

- Pre-dialysis bicarbonate was available and evaluated during model building; it was not retained in the final adjusted LMM after backward elimination. We added a short discussion in the page 9 noting that acid–base status may influence the calcium–PTH set-point and response to calcimimetics, and that residual confounding related to imperfectly captured acid–base physiology cannot be excluded.

It also appears that the authors did not include the dialysis technique as an independent covariate, separate from the dialysate type and dialysate calcium concentration. If it was not significant, I think this should be stated in both the results and the discussion to clearly confirm that the dialysis technique itself did not have an independent effect.

Agreed. Dialysis modality was considered as a fixed-effect covariate among clinically relevant candidates; it was not retained in the final adjusted model after backward elimination. We now state this explicitly in the Results (page 7 in the Analysis of serum PTH Levels Using LMM section) and Discussion to clarify that dialysis technique did not show an independent association with PTH trajectory in the final model specification.

In the limitations, the authors should address the overfitting and potential biases introduced by imputing missing values. A 50% missingness threshold per covariate is somewhat above what is typically recommended. They may rely on the Figure S1 plots to support that the imputed values appear to follow a distribution reasonably similar to that of the original data.

- Agreed. Missingness up to 50% is high and may increase uncertainty and potential bias even under a missing-at-random assumption. We strengthened the Limitations accordingly and refer to Figures S1–S2 (distributional checks and convergence) supporting the plausibility of the imputed values.

Figure S1. The authors should specify that the red line represents the distribution of the observed data, and the black lines represent the distributions of the imputed datasets. It might be helpful to add a brief comment noting that both distributions overlap closely and that the imputed values are consistent with the underlying model. Curves for 25OHD and for BMI presented some problems but this variable was not selected in final model.

- Agreed. We have updated the Figure S1 legend to specify that the red line represents the observed distribution and the black lines represent the distributions of the imputed datasets, and added a brief note indicating close overlap overall.

Figure S2. For the convergence plots, the authors should indicate that the x-axis represents the number of iterations, and they should add a brief comment explaining that convergence was achieved for all variables, with stability across iterations and no drift, thus supporting the validity of the imputation procedure.

- Agreed. We have updated the Figure S2 legend to indicate that the x-axis corresponds to the number of iterations, and added a brief statement that stability across iterations supports convergence of the imputation procedure.

Reviewer #5: The authors have conducted a retrospective study on the effect of dialysate buffer practices on serum PTH levels in patients on maintenance hemodialysis (HD). May I mention the following:
1. The hypothesis of the study seems to be: Dialysate buffer affects serum PTH in HD patients. Serum PTH is mainly affected by calcium, phosphate, FGF 23 AND the treatment of hyperparathyroidism given to the patients. Please give the plausible explanation

- We agree. Our a priori hypothesis is that dialysate buffer may influence PTH trajectories indirectly via calcium balance/ionized calcium, but we explicitly account for major determinants of PTH in the adjusted LMM. Specifically, the model includes repeated serum calcium and phosphate (and other mineral biomarkers) and key SHPT treatments captured in the EHR (calcium salts, active vitamin D/alfacalcidol, and other relevant therapies recorded as time-updated indicators). FGF23 was not available in this real-world dataset and is therefore acknowledged as a potential unmeasured confounder. This was clarified in the limitations of the study in page 11.

2. Please mention pre- and post dialysis serum bicarbonate concentrations

- Pre-dialysis bicarbonate was routinely available and is reported among the monthly biomarkers (including baseline values in Table 1). Post-dialysis bicarbonate was not systematically measured in routine care and was not available in the EHR extract; we therefore clarify in the Methods/Limitations that bicarbonate values refer to pre-dialysis measurements only.

3. To say that the type of dialysate buffer is affecting serum PTH level all above factors would need to be analysed with appropriate statistical analysis

- We agree with the principle. Accordingly, we used a longitudinal linear mixed-effects model with patient-level random effects to analyze repeated PTH measurements and adjusted for baseline PTH and clinically relevant time-updated covariates (including mineral biomarkers, dialysate calcium concentration, and SHPT treatments). Given the observational, non-randomized design and unavailable variables (e.g., FGF23, intradialytic ionized calcium, post-dialysis bicarbonate), we frame our conclusions as associations rather than definitive causal effects.

6. PLOS authors have the option to publish the peer review history of their article ([what does this mean?](https://track.editorialmanager.com/CL0/https:%2F%2Fjournals.plos.org%2Fplosone%2Fs%2Feditorial-and-peer-review-process%23loc-peer-review-history/1/010f019ae2221535-cf4a122f-20bd-44ab-bd24-c205d16739b7-000000/8i0AAHfAJKgXXTbO2TgyFnQQked7so8AzdKwIyVGvSo=239)). If published, this will include your full peer review and any attached files.


If you choose “no”, your identity will remain anonymous but your review may still be made public.


**Do you want your identity to be public for this peer review?** For information about this choice, including consent withdrawal, please see our [Privacy Policy](https://track.editorialmanager.com/CL0/https:%2F%2Fwww.plos.org%2Fprivacy-policy/1/010f019ae2221535-cf4a122f-20bd-44ab-bd24-c205d16739b7-000000/vVb7BJbhzqCTeG6Ci1kgFFzpHYDWLoSRWqcwMWsS2oE=239).

Reviewer #1: No

Reviewer #2: No

Reviewer #3: **Yes:** Krishna Baradhi

Reviewer #4: No

Reviewer #5: No
